# Root-Associated Mycobiome Differentiate between Habitats Supporting Production of Different Truffle Species in Serbian Riparian Forests

**DOI:** 10.3390/microorganisms8091331

**Published:** 2020-08-31

**Authors:** Žaklina Marjanović, Ali Nawaz, Katarina Stevanović, Elmira Saljnikov, Irena Maček, Fritz Oehl, Tesfaye Wubet

**Affiliations:** 1 Institute for Multidisciplinary Research, Belgrade University, Kneza Višeslava 1, 11030 Belgrade, Serbia; 2 Helmholtz Centre for Environmental Research—UFZ, Department of Community Ecology, 06120 Halle (Saale), Germany; ali.nawaz@ufz.de; 3 Faculty of Biology, University of Belgrade, Studentski Trg 3, 11000 Belgrade, Serbia; katarina.stevanovic@bio.bg.ac.rs; 4 Soil Science Institute, Teodora Drajzera 7, 11000 Belgrade, Serbia; soils.saljnikov@gmail.com; 5 Department of Agronomy, Biotechnical Faculty, University of Ljubljana, Jamnikarjeva 101, 1000 Ljubljana, Slovenia; irena.macek@bf.uni-lj.si; 6 Faculty of Mathematics, Natural Sciences and Information Technologies (FAMNIT), University of Primorska, Glagoljaška 8, 6000 Koper, Slovenia; 7 Agroscope, Competence Division for Plants and Plant Products, Ecotoxicology, Müller-Thurgau-Str. 29, 8820 Wädenswil, Switzerland; fritz.oehl@agroscope.admin.ch; 8 German Centre for Integrative Biodiversity Research (iDiv) Halle-Jena-Leipzig, Deutscher Platz 5e, 04103 Leipzig, Germany

**Keywords:** *Tuber* sp., ectomycorrhiza, ITS2, core-mycobiome, fungal metabarcoding

## Abstract

Balkan lowlands bordering with the Pannonia region are inhabited by diverse riparian forests that support production of different truffle species, predominantly the most prized white truffle of Piedmont (*Tuber magnatum* Pico), but also other commercial species (*T.* *macrosporum* Vitt., *T*. *aestivum* Vitt.). Surprisingly, little is known about the native root-associated mycobiome (RAM) of these lowland truffle-producing forests. Therefore, in this study we aim at exploring and comparing the RAMs of three different truffle-producing forests from Kolubara river plane in Serbia. Molecular methods based on next generation sequencing (NGS) were used to evaluate the diversity of root-associated fungal communities and to elucidate the influence of environmental factors on their differentiation. To our knowledge, this is the first study from such habitats with a particular focus on comparative analysis of the RAM in different truffle-producing habitats using a high-throughput sequencing approach. Our results indicated that the alpha diversity of investigated fungal communities was not significantly different between different truffle-producing forests and within a specific forest type, while the seasonal differences in the alpha diversity were only observed in the white truffle-producing forests. Taxonomic profiling at phylum level indicated the dominance of fungal OTUs belonging to phylum Ascomycota and Basidiomycota, with very minor presence of other phyla. Distinct community structures of root-associated mycobiomes were observed for white, mixed, and black truffle-producing forests. The core mycobiome analysis indicated a fair share of fungal genera present exclusively in white and black truffle-producing forest, while the core genera of mixed truffle-producing forests were shared with both white and black truffle-producing forests. The majority of detected fungal OTUs in all three forest types were symbiotrophs, with ectomycorrhizal fungi being a dominant functional guild. Apart from assumed vegetation factor, differentiation of fungal communities was driven by factors connected to the distance from the river and exposure to fluvial activities, soil age, structure, and pH. Overall, Pannonian riparian forests appear to host diverse root-associated fungal communities that are strongly shaped by variation in soil conditions.

## 1. Introduction

Investigations of forest soil-inhabiting fungal communities have benefited from the advances in next generation sequencing (NGS) techniques that enabled fast and precise analyses of taxa originating directly from environmental root or soil samples [1,2]. A large number of studies reported that, under natural conditions, several hundred fungal species are associated with plant roots in different forest ecosystems [3,4,5,6]. The taxonomic structures of these fungal assemblages are driven by abiotic factors i.e., soil water and oxygen content, pH and C/N ratio, as well as biotic factors, i.e., vegetation types and other co-existing microbial taxa [1,7,8,9,10]. Soils of temperate forests are inhabited by a wide range of seasonally-varying fungal communities, usually dominated by Basidiomycota, followed by Ascomycota, and to a lesser extent other phyla (Chytridiomycota, Glomeromycota, Mortierellomycota) [2,11,12,13], all having distinct ecological functions that are represented by different trophic groups: symbiotrophic (endophytes, mycorrhiza), pathotrophic, and saprotrophic fungi [6,14]. The majority of published data on European forest mycobiomes originate from temporal and boreal regions, where the soil, climatic, and vegetation conditions are not so variable [10,12,13,15]. However, studies on the forest mycobiomes of the sub-Mediterranean zone, especially the Balkan Peninsula, characterized by spatially varying and very diverse biotic and abiotic conditions are, in general, very scarce [6,16], while those from ecologically-specific ecosystems (e.g., European riparian forests) are completely missing.

The Balkan Peninsula, the easternmost of Europe’s three large southern peninsulas is a biodiversity rich area bordering with the wide Pannonia basin in the north through territories of Croatia, Serbia and Romania. This border is not only geographical line between Central and South Europe, but also a borderline between different terrain elevations, geological formations of different age and origin as well as climatic zones and floristic regions (Pannonian to sub-Mediterranean) [17]. Since the Holocene climatic era started to dominate, this is where forest-steppe plains have been meeting forest inhabited hills before the humans transformed it into the agricultural area, why the original forests turn out to be on the edge of extinction [18]. As the mountains that feed sediments of these bordering regions are mostly calcareous (easternmost parts of Dinaridi), the soils formed in their foothills are very fertile and usually neutral to slightly acidic. The natural vegetation types are riparian forests [17,19,20], which are strongly influenced by the fluvial actions that are washing down these sediments from the mountains. The dominant tree species are mainly ectomycorrhizal (ECM): *Quercus robur*, *Populus alba*, *P. nigra*, *Tilia* spp. and *Carpinus betulus*, but also include arbuscular mycorrhizal (AM) tree species: like *Ulmus* spp., *Acer* spp, *Fraxinus* spp. and *Crategus* spp. These trees are capable of forming biomass-mighty communities, that are defined by the level of fluvial activity/groundwater vertical dynamics and soil structure [21]. Due to the bulky and diverse shrub and herbaceous layers, riparian forest ecosystems are characterized by high plant species richness, density and productivity [21,22,23,24], which is usually not expected for communities dominated by ECM trees [25]. In contrast to Northern European regions, riparian forests in southern borderline of Pannonia support production of different true truffle species of commercial value [26]. The dominant sporocarp producing species in such ecosystems is the world’s most prized and the most endangered truffle species i.e., *Tuber magnatum* Pico [26,27]. Unlike some other truffle species, so far, this cannot be produced in artificially formed plantations of inoculated trees, which is why the fast disappearing natural habitats are the only sources for obtaining its ascocarps. Additionally, three other commercial truffle species (*T. aestivum* Vitt., *T. macrosporum* Vitt and *T. brumale s.l.* Vitt) can also be collected from these habitats, as well as some non-commercial species like *T. rufum* Vitt, *T. foetidum* Vitt, *T. excavatum* Vitt, while above-ground sporocarps of ECM species appear very rarely [26].

The species of the highest interest, *T. magnatum* producing habitats are old forests dominated by ECM trees, situated on sites with ubiquitous soil water supply [28,29]. Abiotic factors that influence such habitats have been recently described [27,30,31,32,33], as well as truffle mycelia distribution connection to soil conditions [29,33] but, in general, very little is known about root inhabiting fungal communities and their potential ecological roles in truffle habitats. Previous studies have focused on ECM fungal partners of trees using morphotyping and genotyping of ECM rootlets isolated from sampled soil cores [34,35,36,37,38,39]. Even though not very abundant, ECM of some truffle species can be detected in natural habitats [40], while *T. magnatum* ECM are almost impossible to find in the field [38,39,41], even though its mycelium is widespread in truffle-producing soils [33,42].

To achieve a first holistic overview of root-associated mycobiomes (RAM) in truffle-producing riparian forest ecosystems in borderline with the Pannonia basin and Balkan Peninsula, we studied three different, but closely positioned, truffle-inhabiting forests located in the floodplain in Northwest Serbia. We analyzed fungal communities of root samples collected in soil cores from three forest types located at different distance from the river bank. The sampled forest types support the production of different truffle species (white truffle: *T. magnatum*; autumn black truffle: *T. macrosporum*; and mixed: both of those + summer truffle *T. aestivum*). Paired-end Illumina MiSeq sequencing of ITS2 fragment of the fungal ITS rDNA was performed to: (1) assess the impact of dominant truffle-producing forest type on the root-associated fungal community composition, (2) explore temporal patterns of fungal communities in the respective forest types, (3) identify the biotic and abiotic parameters shaping the root-associated fungal community composition, and (4) identify the dominant fungal functional groups among the three forest types. Thus, we tested the hypotheses that the root-associated fungal communities show temporal patterns and their composition is associated with the conditions supporting production of dominant truffle species in the particular forest type. Furthermore, as all the three forest ecosystems are dominated by ectomycorrhizal tree species, we assumed the fungal communities to be dominated by symbiotrophs, mainly ECM fungi, regardless of the forest type and the studied forest types are characterized by low core-mycobiome.

## 2. Materials and Methods

### 2.1. Study Area

The area of the Kolubara river basin in Western Serbia was chosen due to its closeness to the easternmost hills belonging to Dinaric massive and not so wide fluvial influence. The climate is characterized as continental with Mediterranean influences. Data collected from the nearest meteorological station (Valjevo) showed an average yearly precipitation of 787.4 mm and average yearly temperature of 11.4 °C. The warmest and coolest months are July and January, with an average temperature of 21.9 °C and 0.6 °C, respectively (http://www.hidmet.gov.rs). The Kolubara basin has a continental pluviometric regime that is characterized by a maximum rainfall at the beginning of summer (May/June) and a minimum in winter [43].

Three habitats were chosen for sampling due to the previously known history of truffle production (received from highly experienced truffle hunters group and based on evidence collected during the last 15 years, A. Glišić, personal communication): (1) degraded *Populus alba* dominating forest on the river bank (national forest classification type 14410, “*Populetum albae*, on recent alluvial sediments”) occasionally producing only *T. magnatum* in wet years (44°21.48″ N 20°09′20″ E, app. 3+ m distance from the river, 111 meters above sea level( masl)) here defined as “white truffle-producing forest”; (2) *Quercus robur* dominating forest (national forest classification type 15,330 “*Carpino-Fraxino-Quercetum roboris caricetosum remotae,* on semiglay soil type in area not prone to regular flooding” abundantly producing *T. magnatum* and occasionally *T. macrosporum* in autumn and *T. aestivum* in late summer in all years (44°21′02″ N 20°11′06″ E, app. 750 m distance from the river on 115 masl) here defined as “mixed truffle-producing forest”, and (3) *Tilia cordata, Carpinus betulus*, and *Quercus robur* mixed forest (national forest classification type 16,140 “*Tilio-Carpino-Quercetum roboris*, in valleys, on deluviums not exposed to fluvial activity”) occasionally producing only *T. macrosporum* in wet years (44°17′24″ N, 19°59′12″ E, app. 1500 m distance from the river, 156 masl), here defined as “autumn black truffle-producing forest”. Mixed truffle and black autumn truffle-producing forests were near to natural, e.g., timber harvesting has been performed but the structure of the stands and canopy cover remained preserved.

### 2.2. Sampling of Roots and Soil

In each investigated forest type, 9 m^2^ (3 × 3 m) plots spaced approximately 10m apart in previously marked truffle productive areas, were sampled in three different seasons (June, August and November) in 2016. According to Serbian Institute for Hydrometeorology, this was a year with above average rainfall (http://www.hidmet.gov.rs) and all investigated sites produced truffles in the previously described manner (A. Glišić, personal communication). The seasons/sampling times were chosen according to the most expressed differences in soil water content (which appeared to determine nutrient availability and therefore probably root-fungal associations) during the vegetation period, which is highest in June, lowest in August and average in November (in winter and spring the soils in explored habitats are either frozen, or water saturated, or inundated) [27]. Three soil cores (excluding organic layer, 10 cm width × 15 cm depth) with roots were sampled from each plot in each season and stored at 4 °C until processing (within 1–2 days), resulting in 27 samples. Soil cores were taken within the chosen plots in the triangle formation spots and with app. 30 cm distance from previous points every sampling time. All roots that did not look strongly lignified were extracted from the soil cores, rinsed under tap water to remove non-adherent soil, dried at 70 °C and stored at −20 °C until further DNA extraction to assess the root-associated fungal community that is hereafter named “root associated mycobiome” (RAM).

### 2.3. Soil Analyses

Soil from the sampled plots to be used for the analyses was dried at ambient temperature and kept at 4 °C. Soil pH was determined with a glass electrode pH-meter in 1:2.5 water solution. Available P_2_O_5_ and K_2_O were determined according to Enger and Riehm [44], where 0.1 N ammonium lactate (pH = 3.7) was used as an extractant. After the extraction, K was determined by flame emission photometer and P by spectrophotometry after color development with ammonium molibdate and SnCl_2_. Total C and N were analyzed with a total CNS analyzer VARIO EL III (ELEMENTAR Analysensysteme GmbH, Hanau, Germany) [45]. Since it has been previously detected as the factor strongest connected to seasonal variability of the soil environment with the season in similar habitats, available P_2_O_5_ was measured in all seasons, while all other soil parameters that are assumed as stable were measured in autumn samples (when the truffle production occurs) [27].

### 2.4. DNA Isolation and Illumina Sequencing

Dry root samples from each plot/season were grinded to fine particles using sterile mortar and pestle. DNA was isolated from 0.5 g of the root material, in two replicates from every plot/season sample using PowerPlant DNA Isolation Kit (MO BIO Laboratories, Carlsbad, CA USA) following the manufacturer’s instructions. Subsequent PCR amplifications and paired-end Illumina sequencing was performed at Microsynth AG (Balgach, Switzerland). Amplicon libraries were prepared in a semi-nested PCR protocol to amplify fungal ITS2 region using the primer combinations ITSF1, fITS7 [46] and ITS4 [47]. The first PCR reaction mix included DNA template, 7.5 pmol of each forward and reverse primer (using the primer pairs ITSF1 and ITS4) and 0.5 U of KAPA HiFi HotStart (KAPA Biosystems, Cape Town, South Africa) in 25 µL final volume. The amplification was carried out for 20 cycles using the following parameters: 15 min 95 °C pre-denaturation; 98 °C for 30 s, 50 °C for 60 s, 72 °C for 60 s and a final extension at 72 °C for 10 min. In the second PCR, 5 µL of amplified product form the first PCR was used as template in a final reaction volume of 25 µL containing 8 pmol of both forward and reverse primers (using the primer pairs fITS7 and ITS4) and 0.4 U of KAPA HiFi HotStart (KAPA Biosystems, Cape Town, South Africa). This amplification was carried out for 15 cycles using the following parameters: 3 min 95 °C pre-denaturation; 98 °C for 20 s, 50 °C for 30 s, 72 °C for 30 s and a final elongation at 72 °C for 5 min. The final ITS2 library was sequenced on Illumina MiSeq using a v3 chip (2 × 300 bp) and 600 cycles. Afterwards, the reads were demultiplexed using Illumina Realtime analysis software to sort the reads into their original samples using the barcodes from the adaptors.

### 2.5. Bioinformatics Analysis

The paired-end sequences generated by Illumina MiSeq were processed to extract high quality reads by using MOTHUR [48] and OBI Tools [49] software suits as previously explained in [50,51]. Briefly, forward and reverse raw reads were assembled using the simple-Bayesian algorithm with a minimum overlap of 20 nucleotides as implemented in PANDAseq (v.2.8.1) [52]. All assembled reads were then filtered according to the following thresholds: (i) minimum average Phred score of 26, (ii) no ambiguous base in the whole read length, and iii) maximum length of 10 homopolymers. Chimeric sequences were removed using the UCHIME algorithm [53] as implemented in MOTHUR. At a threshold of 97% sequence similarity, the reads were then clustered into operational taxonomic units (OTUs) using the VSEARCH algorithm [54]. The representative sequences for each OTU were taxonomically assigned against the reference sequences from the UNITE database (version v.7.0) [55] using naïve Bayesian classifier [56] as implemented in MOTHUR using the default parameters. After clustering of sequences into OTUs, the sequences were further quality filtered using ITSx (v. 1.0.11) [57] to remove 5.8S and 28S fragments and any non-fungal reads from the dataset. Fungal OTUs detected in three different truffle-producing forests were functionally annotated against FUNGuild database to get meaningful ecological and functional categories which included the confidence ranking of highly probable, probable, and possible [58]. We used “highly probable” and “probable” confidence ranking for trophic mode and functional guild assignment. The taxon with confidence ranking of “possible”, more than two functional guild assignments were grouped as “uncertain” [58]. The fungal ITS2 raw sequence dataset is deposited in the National Center for Biotechnology Information (NCBI) Sequence Read Archive (SRA) and is available as bioproject number PRJNA578132.

### 2.6. Statistical Analysis

All statistical analysis of the data was performed in R software [59]. To remove the inherent sequencing bias between the samples due to varying number of reads, the OTU matrix was randomly normalized to the smallest number of reads per sample to get minimum common sequencing depth. Rare OTUs (single-, double-, and tripletons) which might have been originated by the artificial sequences [60] were removed from the dataset. In order to assess the effect of removing rare OTUs from the dataset, we performed a Mantel test using Bray-Curtis dissimilarities with 999 permutations to assess the correlations between the matrix including rare OTUs and the matrix excluding the rare OTUs. The results indicated that the removal of rare OTUs have no effect on the fungal community composition (R_Mantel_ = 0.999, *p* = 0.001). Therefore, for further statistical analysis the dataset without rare OTUs was used. Sample based rarefaction curves and alpha diversity indices (species richness and Shannon diversity index) were calculated using vegan package [61] implemented in R. The comparison of the alpha diversity was tested using one-way ANOVA with further pairwise comparisons using Tukey’s HSD test (*p* < 0.05). To measure the distance between communities, we used Bray-Curtis dissimilarity distance matrix and non-metric multidimensional scaling (NMDS) to visualize the separation of communists in two-dimensions using vegan package. Significant (*p* < 0.05) environmental variables and fungal genera were fitted to the NMDS ordination plots using the Goodness-of-fit statistics (*R*^2^) calculated using the “envfit” function in the vegan package, with *P* values based on 999 permutations. To check the significant differences between the communities, we used two-way PERMANOVA (permutations = 999) using the *adonis* function of the vegan package with the truffle-producing forest type and sampling season as explanatory variables.

## 3. Results

### 3.1. Soil Characterization

The analyses of soil parameters revealed significant differences in soil types and structure in explored habitats within explored forest types (Table 1 and Supplementary Table S1). Briefly, according to the National Soil Map of Serbia, our results and WRB 2014 Soil Classification, soil in white-truffle-producing forest was classified as Calcaric Fluvisol, with high pH, very poor in N and SOM, but very high C/N ratio and moderately supplied with P. Mixed truffle productive forest soil was classified as Fluvic Gleysol with high clay content, slightly acidic to neutral pH, very high N supply, moderate SOM content, and low C/N ratio, while P availability strongly varied with the season. Autumn black-truffle-producing forest soil was classified as Dystric Cambisol, with low pH, high SOM content, high N supply, low C/N ratio and low P availability. In all soils K was high and obviously not the limiting factor for plant growth.

### 3.2. Overview of Sequencing Dataset

We obtained a total of 5,851,606 raw reads resulting in 5,310,300 paired-end reads from Illumina Paired-end sequencing of the fungal ITS rDNA from the 27 root samples, 9 representing each of the three truffle-producing forest types across three sampling points and periods. Sequential bioinformatic filtering of low-quality, short read length, chimera and non-fungal reads resulted in a total of 944,634 reads, which were retained for further analysis. The total number of reads varied between samples within the range of 3763 and 82,142. Therefore, the dataset was rarefied to 3750 reads per sample to achieve a similar sequencing depth for all the samples representing 2243 fungal OTUs at 97% sequence similarity. Sample-based rarefaction curves of OTUs at 97% sequences similarity from all the samples are provided in supplementary information Figure S1.

### 3.3. Alpha Diversity and Community Composition of Root-Associated Mycobiome in Different Truffle-Producing Riparian Forest Types

**Figure 1:**
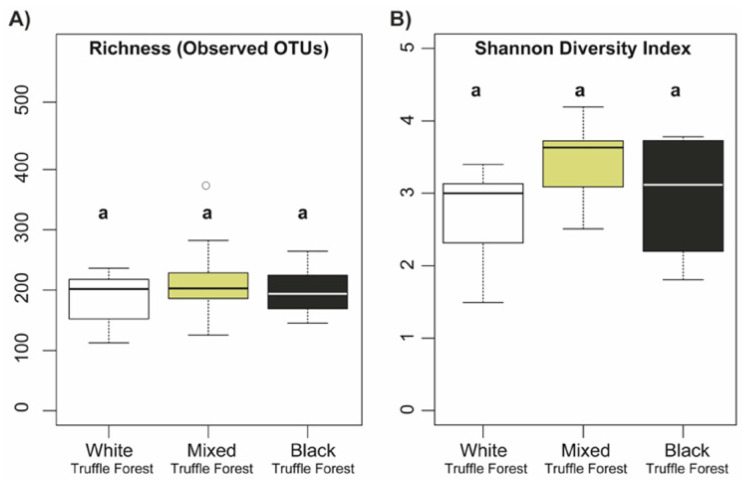
Observed richness (number of OTUs) (**A**) and Shannon diversity index (**B**) of root-associated mycobiome in white, mixed, and autumn black truffle-producing forest in Serbian riparian forests. Significant differences in observed richness and Shannon diversity Index between different truffle-producing forests were calculated using one-way ANOVA followed by Tukey’s HSD test (*p* > 0.05).

**Figure 2:**
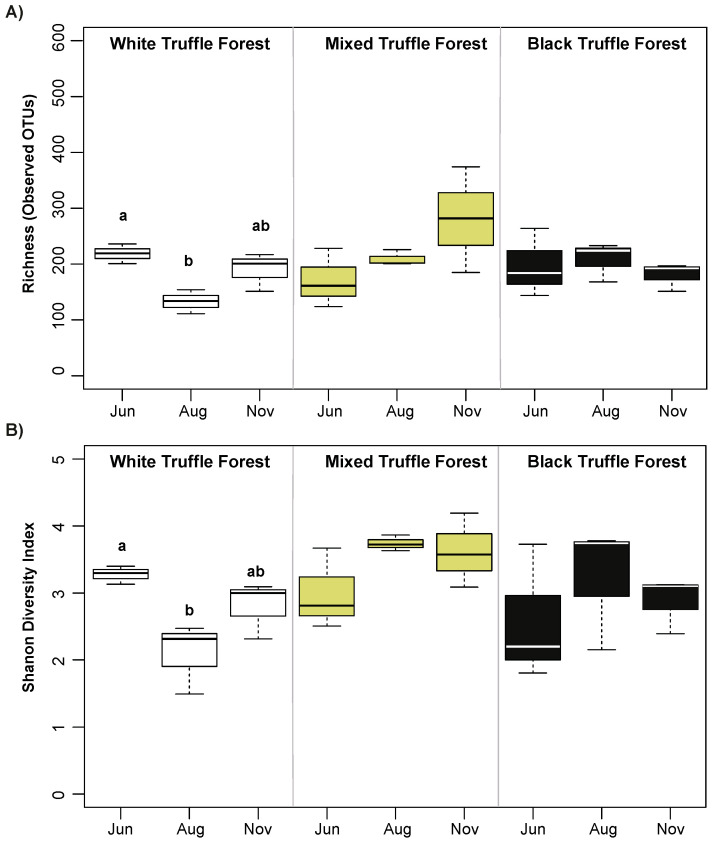
Observed richness (number of OTUs) (**A**) and Shannon diversity index (**B**) of root-associated mycobiome in white, mixed, and autumn black truffle-producing forest across three different sampling months (June, August, and November) in Serbian riparian forests. Significant differences in observed richness and Shannon diversity Index between different truffle-producing forests were calculated using one-way ANOVA followed by Tukey’s HSD test (*p* > 0.05).

We found a total of 1384 fungal OTUs (after removal of rare taxa i.e., single, double and triple -tons) across all 27 samples. One-way ANOVA followed by pairwise comparisons of different truffle-producing forest types using Tukey’s test revealed that the fungal alpha diversity measures (richness measured as observed OTUs and Shannon diversity index) were not significantly different (ANOVA *p* > 0.05) between white, mixed and black truffle-producing forests (Figure 1A,B), whereas, a significant seasonal variation (ANOVA *p* < 0.05) in fungal alpha diversity (richness and Shannon diversity) was observed in the white truffle-producing forest between June, August, and November (Figure 2A,B). In contrast to white truffle-producing forest, no significant seasonal variations were observed in fungal alpha diversity of mixed and autumn black truffle-producing forests.

**Figure 3:**
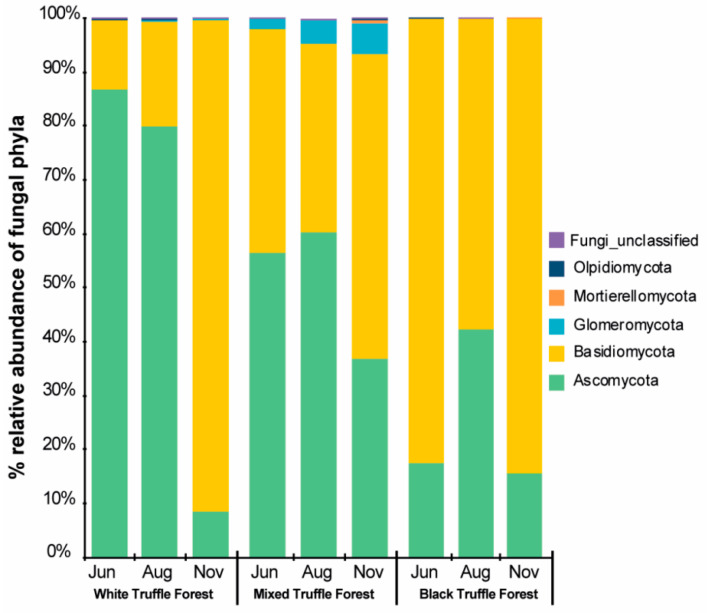
Relative abundance of root-associated fungal phyla of white, mixed, and black truffle-producing habitats across three different sampling months in Serbian riparian forests.

Overall, the root-associated mycobiome of three different forest types was mainly dominated by the fungal OTUs from phylum Basidiomycota (56%) and Ascomycota (42%), followed by small fractions of OTUs from Glomeromycota (2%). Keeping different forest types in account, there were variations in the dominant fungal phyla (between different sampling seasons (June, August, and November) (Figure 3). Specifically, white truffle-producing forest in June and August was dominated by the members of phylum Ascomycota (87% and 80%, respectively), whereas, in November, members of phylum Basidiomycota dominated the fungal community (91% Basidiomycota and 9% Ascomycota). In the mixed truffle-producing forest, the fungal community was represented by the members of phylum Ascomycota (55% in June, 60% in August, and 37% in November) and phylum Basidiomycota (41% in June, 35% in August, and 57% in November). Noteworthy, the members of phylum Glomeromycota appeared only in the mixed truffle-producing forest across all the sampling seasons (2% in June, 4% in August, and 6% in November). In contrast to white truffle-producing forest, the autumn black truffle-producing forest except in August, was dominantly represented by the members of phylum Basidiomycota (82% in June, 84% in November, and 58%in August) (Figure 3).

### 3.4. Beta Diversity and Correlation between Root-Associated Mycobiomes and Environmental Physicochemical Variables

**Figure 4:**
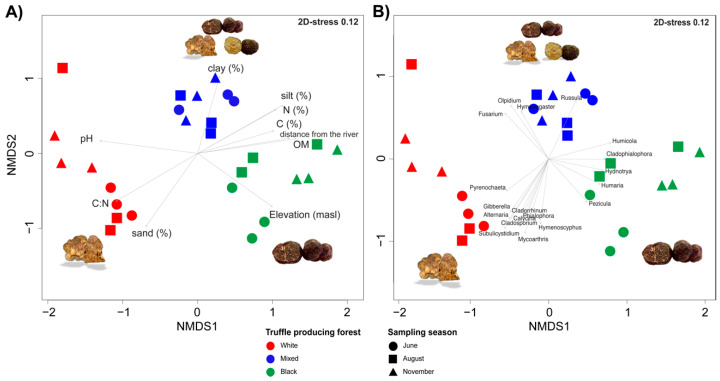
Two-dimensional non-metric multidimensional scaling (2D-NMDS) ordination of root-associated mycobiome of white (red), mixed (blue) and autumn black (green) truffle-producing forests in Serbian riparian forests in three different sampling times (June (circles), August (squares), and November (triangle). The NMDS ordination was fitted with soil physiochemical parameters (**A**) and with fungal genera (**B**) (only significant soil physiochemical parameters and fungal genera *p <* 0.05 are shown).

The fungal community structure of the different truffle-producing forests is presented in two-dimensional non-metric multidimensional scaling (2D-NMDS) ordination plot (Figure 4). In the NMDS ordination, samples from the same forest type appeared to be in close proximity compared to the samples from other forest types which indicated that distinct fungal communities are associated with the three truffle-producing forests. Consistent to this, the analysis indicated that the fungal community structure differed significantly between white, mixed and black truffle-producing forests (PERMANOVA *F * =  4.8, *df*  =  2, *p * =  0.001), and across different sampling months (PERMANOVA *F * =  2.12, *df * =  2, *p * =  0.001) with a significant interaction between the forest type and sampling season (PERMANOVA *F * =  2.1, *df * =  4, *p*  =  0.001). The Goodness-of-fit statistics indicated that different set of environmental physicochemical variables (Table 2) and the geographical locations of the forest types were significantly correlated with the community structure of different truffle-producing forest (Figure 4A). For instance, soil pH, C:N ratio and sand content were positively correlated with the fungal community of white truffle-producing forest. Whereas, the amount of the organic matter, carbon and nitrogen content of the soil, distance of the sampling plots from the river and the altitude were significantly correlated with the fungal communities from black truffle-producing forest (Figure 4A, Table 2).

Apart from different environmental parameters, certain fungal genera represented by six fungal families (i.e., Cladosporiaceae, Discinaceae, Helotiaceae, Hyaloscpyhaceae, Olpidiaceae, and Russulaaceae) significantly correlated (*p* < 0.01) with three forest types (Figure 4B, Table 3). Specifically, white truffle-producing forest was positively correlated with the genera *Pyrenochaeta* sp., *Gibberella* sp., *Alternaria* sp., *Subulicystidium* sp., *Cladorrhinum* sp., *Phialophra* sp., and *Hymenoscyphus* sp. The genera *Fusarium* sp., *Olpidium* sp., *Hymenogaster* sp., and *Russula* sp. were significantly correlated with the mixed truffle-producing forest, while *Humicola* sp., *Cladophialophora* sp., *Hydnotrya* sp., *Humaria* sp., and *Pezicula* sp. were significantly correlated with autumn black truffle-producing forest.

### 3.5. Core Mycobiome Across Forests Types and Sampling Seasons

**Figure 5:**
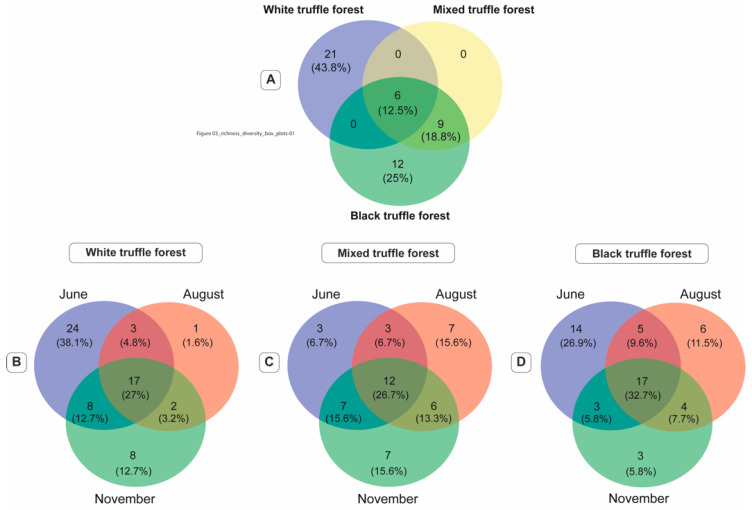
Venn diagrams illustrating shared and specific core fungal genera (defined as genera detected in 95% of the samples of specific forest types) in white, mixed, and black truffle-producing forests (**A**) and within each truffle-producing forest type, across three different sampling time-points (June, August, and November, (**B**–**D**), respectively). The numbers and percentage values in each Venn diagram represents the number of fungal genera and their percent distributions respective forest type (**A**) or sampling months (**B**–**D**).

The core mycobiome (defined as taxa present in 95% samples of the specific forest type) of the white, mixed and black truffle-producing forest is represented by 27, 15 and 27 fungal genera respectively (Table 4). The fungal genera associated with individual core mycobiomes were represented by 69.7%, 32.45% and 39.55% of the total sequences of the white, mixed and black truffle-producing forest respectively (Table 4). The Venn diagram in Figure 5A revealed that 12.5% of the core mycobiome (6 genera) was shared between the white, mixed and black truffle-producing forests. Interestingly, 43.85% (21 genera) and 25% (12 genera) of the core mycobiome of the white and autumn black truffle-producing forests, respectively, were unique. The autumn black and mixed truffle-producing forests shared 18.8% (nine genera) of their core mycobiomes, whereas there was absolutely no fraction of the core mycobiome was shared between white and mixed truffle forest (Figure 5A). Considering the seasonal patterns within the investigated forests, a consistent fraction of core mycobiome (17–27%) was shared between the three sampling seasons in the respective truffle-producing forests (Figure 5B–D).

### 3.6. Distribution of Fungal Trophic Modes and Functional Guilds

**Figure 6:**
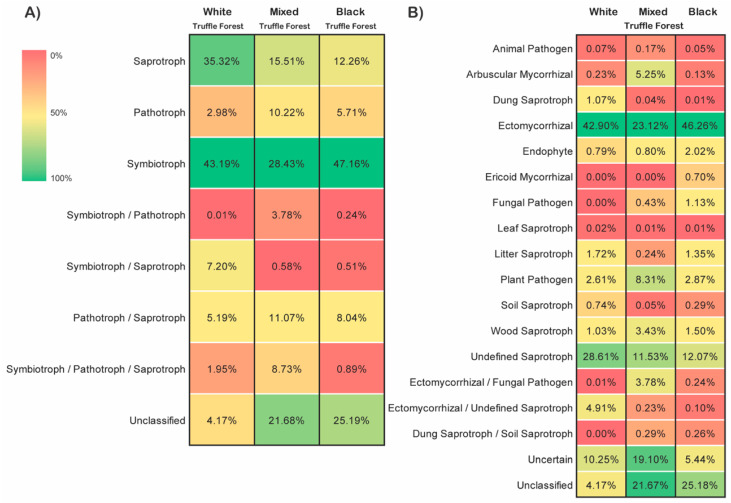
Relative abundance-based heat-maps of the fungal trophic modes (**A**) and functional guilds (**B**) inferred by FUNGuild in white, mixed and black truffle-producing forests in Serbian riparian forests. The values in each color block represents the relative abundance (%) of the respective trophic mode or functional guild.

**Figure 7:**
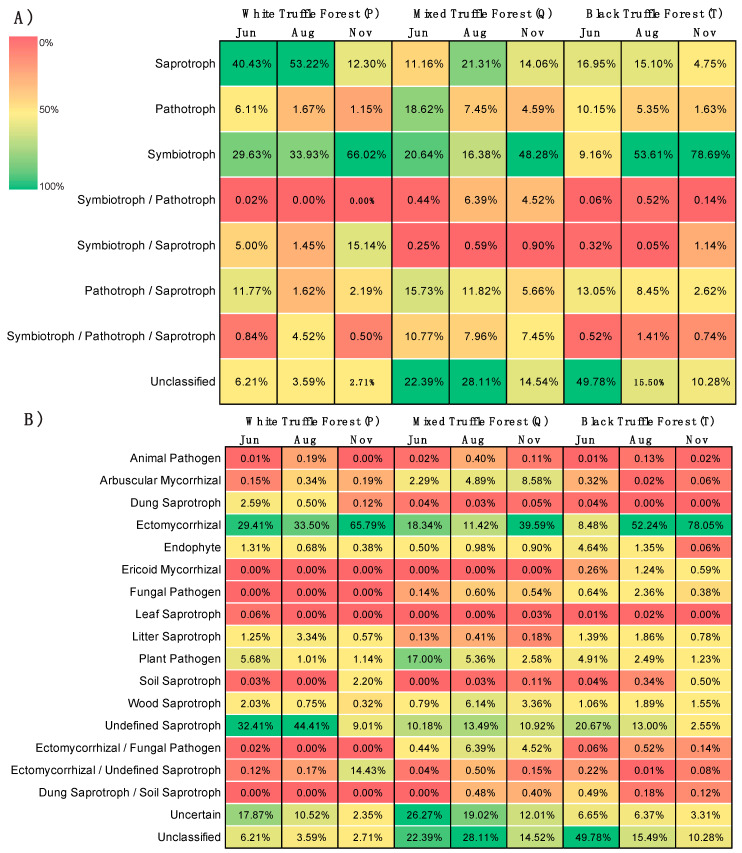
Relative abundance-based heat-maps showing seasonal variations in fungal trophic modes (**A**) and functional guilds (**B**) inferred by FUNGuild in white, mixed, and black truffle-producing forests in Serbian riparian forests. The values in each color block represents the relative abundance (%) of the respective trophic mode or functional guild.

Based on functional guild and trophic mode analysis, we found that in all the three forest types, symbiotrophs dominated the fungal community with 43.19%, 28.43%, and 47.16% in white, mixed and black truffle-producing forests respectively (Figure 6A). The saprotrophs were the second abundant fungal trophic groups (Figure 6A). We also detected a smaller proportion of the pathotrophs within the community. Further analyses of fungal functional groups revealed that within symbiotrophs, the community is dominated by ectomycorrhizal fungi (42.90%, 23.12% and 46.26% in white, mixed, and black truffle-producing forests respectively) (Figure 6B). The distributions of fungal trophic modes and the ecological guilds across three sampling months (June, August and November) in each forest types were also fairly consistent with that of the variation observed among forest types (symbiotrophs being the dominant trophic mode and ectomycorrhizal fungi the dominant functional group) (Figure 7). However, we also observed the strong temporal dynamics between saprotrophs and symbiotrophs in white truffle-producing forest (Figure 7A).

## 4. Discussion

In this contribution we presented distinct root-associated mycobiomes of three forest types positioned along the alluvial plane environmental gradients in North-west Serbia characterized by a distinct pattern of truffle species production. Truffles as ECM fungi that produce ascocarps known for nutritional, aromatic and economic significance, were the objects of previous investigations aiming to elucidate communities of ectomycorrhizal fungi associated with specific truffle production, or to detect/identify truffle species [36,37,38,41,62]. Here we go a step forward, presenting a comparative analysis of overall root-associated fungal communities of three different truffle-producing natural habitats positioned in the close proximity (same mesoclimatic conditions), along the environmental gradient caused by the different hydrological regimes. In two of these habitats, truffle production has been detected occasionally during the last 15 years period, marking them as suboptimal truffle habitats (forest on the river bank and forest on the alluvial terrace), while forest situated on the middle of the alluvial plane produced significant amounts in all previously recorded years and can be signed as ideal habitat for *T. magnatum*, but also producing *T. aestivum* and occasionally *T. macrosporum*. Since the entire root-associated mycobiome of truffle-producing habitats have never been explored before, only indirect comparisons can be made with studies in the related environmental settings in different habitats [34,35,36,37,38,39].

### 4.1. Root-Associated Fungal Communities Are Related to the Environemntal Conditions Supporting Production of Dominant Truffle Species

Similar to large scale study of RAM in European temperate forests [13], we have not observed significant differences in fungal alpha diversity across the investigated forest types. The methodologies used here and that of Schroter et al. [13] were not comparable, but it is worth observing that overall numbers of detected OTUs appeared much higher in Balkan riparian forests than in European temperate forests, even though our number of investigated plots, and the areas of investigation were much smaller. The observed non-significant differences in the fungal alpha diversity and significant differences in the taxonomic composition indicates the fact that the studied different truffle grounds are equally rich in terms of fungal OTUs but harbor diverse taxonomic groups. Such taxonomic differences in the fungal communities point towards selective recruitment of the root-associated fungi determined by the host truffle grounds. Multiple studies showed that host species identity is an important factor in structuring ectomycorrhizal fungal communities in various ecosystems [3,63,64]. However, recent investigations revealed that environmental filtering also plays a dominant role in structuring both free-living and symbiotic fungal communities at fine spatial scales [13,65]. Glassman et al 2017 [13,65] documented that pH and organic matter primarily influenced total soil fungal communities in closely positioned forest ecosystems, while pH and cation exchange capacity (not host species) were the major factors affecting EMC community composition. The environmental factors not only play roles in shaping the root-associated fungi, but also dictate the production of specific truffle species [30,66,67,68,69,70]. For instance, white truffle (*T. magnatum*) requires specific seasonal dynamics of soil water content in order to complete its life cycle but, in general, it demands constant presence of certain water content in the soil [27,29].

Unlike our study, the previous related studies on fungal (ECM) diversity of different truffle grounds [34,36,37,38,39] did not include any data on soil physicochemical characteristics, which made it impossible to analyze the influence of soil characteristics on the diversification of symbiotic fungal communities in such habitats. The present study unambiguously showed that soil environments supporting different truffle production play significant roles in shaping the general root-associated fungal communities in closely positioned forests in West Serbia. While availability of K and P appeared not to be differentiating factors for RAMs (Table 2). Large K availability and P unavailability were previously connected to truffle productivity [27], but in our investigation high K availability was probably related to the soil characteristics of the entire investigated alluvial territory, while P availability expressed high variability within and between the forests (Table 1), which may be why the statistical analyses could not relate it to the variation in RAM.

Fluvial activities were also found to play a major role in shaping the root-associated fungal communities through a soil/vegetation gradient (Figure 4A). From the NMDS graph it is obvious that high pH, C:N ratio and sand content (indicators of the direct fluvial activity and active sediment enrichment), were the factors that structured RAM of white truffle-producing forest (Figure 4B). These factors were negatively correlated to the distance from the river and, therefore, the factors that structured RAM of two other forests (Figure 4A). On the opposite side of the alluvial plain (expressed through the variability in elevation), very different RAM was shaped by the low pH, low sand content and high SOM (e.g. the absence of the fluvial activity and an active river brought sediment enrichment) in the autumn black truffle-producing forest. Acidification of the top soil layer probably occurred through the eluviation processes that transferred Ca to deeper ancient alkaline sediments (Supplementary Table S1b). The anticipated existence of the alkaline sediments in the deeper soil layers has probably supported *T. macrosporum* production even in such low pH measured in the top soil of this site (Supplementary Table S1b), which has never been recorded before. Fine sediments (high clay and silt content) and high N pool are normally characteristic for middle, calm parts of alluvial plains [24]. Together with relatively high pH (maintained by the activity of groundwater vertical movements and consequent Ca enrichment), these variables strongly differentiated RAM of mixed truffle-producing forest, providing highly productive environment for different truffle species (Figure 5A, [26,27]).

### 4.2. Root-Associated Fungal Communities in Balkan Riparian Forests Are Charcaterised by Core and Forest Type Specific Genera

Root-associated fungal communities in forests on the river bank (producing white truffles) were highly differentiated from those in truffle-producing forests on the plane terraces (Table 4). Only six genera were detected in the core mycobiome common for all three forest types, including generalist ECM genera *Inocybe* sp. and *Tomentella* sp., both common constituents of the ectomycorizome in truffle habitats as well as root pathogens and saprotroph/endophytes (Table 4, [71,72]). Mycorrhizal networks appear to have modular nature where certain species associate more often with each other [1,73,74] and this explains the common presence of certain ECM fungi in truffle habitats [72]. Additionally, the spatial structure of fungal symbiotic communities may follow that of host plant roots [75] and plant species composition [76], explaining the differences between explored RAMs. However, *Tuber* sp. appeared as member of the core mycobiome only in white truffle-producing forest, being the only ECM genera apart from generalist *Hebeloma* sp. that has also commonly been detected in truffle habitats [71,72]. Even though truffles are the major fructifying fungi in all studied habitats, none of the highly productive truffle species was detected in RAMs of explored forests with methods applied here. However, other truffle species that have been previously observed as rare in ascocarp surveys from lowland habitats of Serbia [26] were detected in this study, i.e., *T. brumale* Vitt, *T. rufum* Picco, *T. foetidum* Vitt, *T. maculatum* Vitt., and *T. excavatum* Vitt,. All these species (otherwise common in different habitats in majority of Europe) belong to different clades within a genus *Tuber* sp., while highly productive species belong to two other distinct clades (*T. aestivum* and *T. macrosporum* clades) [77]. It may happen that specific protocols should be used when studying root mycobiomes of the truffle habitats, in order to amplify members of all clades, as previously suggested [40].

Interestingly, apart from those shared by all three forest types, there were no other core fungal genera shared either between white and mixed, or between white and black truffle-producing forests (Table 4, Figure 5A). Nine additional genera were shared between black and mixed truffle-producing forests (Table 4, Figure 5A), implying that the white and black truffle forests are characterized by a set of specific root-associated fungal communities, while the fungal communities detected in the mixed truffle-producing forests are subsets of the black truffle forest. Since it has minimum shared core fungal genera (only 6) with mixed and black truffle forests, and highest core genera (21), white truffle forest appeared to support very specific fungal communities as compared to other investigated forests (Table 4). The observed differences in RAMs of white truffle-producing forests was similar to the results of Leonardi et al 2013 [38], who detected very dissimilar ectomycorrhizomes (even on the family level) in four ecologically different habitats of *T. magnatum* in Italy, though the genera that they have recorded appeared in our study as well (Table 3). Black truffle species may be more closely associated with the specific RAMs (Table 4, Figure 5) [35,78,79]. Li, et al. [80] studied the effects of chines black truffle (*T. indicum*) on the ectomycorrhizosphere and endoectomycosphere microbiome of the host tree (*Quercus aliena*) and found that the presence of truffle changed the microbial biodiversity in ectomycorrhizae and ectomycorrhizosphere soil. Core genera specific for autumn black truffle-producing forests (ECM forming *Russula* sp. and *Sebacina* sp., as well as endophytes like *Cladophialophora* sp., *Humicola* sp., *Mycena* sp., *Mortierella* sp.) were commonly detected in ectomycorizomes of black truffle-producing habitats [72,81]. However, *Cennococum* sp., *Entholoma* sp., *Phallus* sp., which were common in autumn black truffle-producing soils are assumed to be more frequent in dry or acidic soils [13,72].

On the other hand, fungal genera specific to white truffle-producing forest were only saprotrophs, endophytes, plant pathotrophs or capable of changing trophic mode in different conditions, which is a common situation for poplars [82]. This habitat has been exposed to constant direct influence of river fluvial activity through flooding events, sedimentation of soil particles and organic debris, as well as abrupt changes of soil water content [83]. Soil conditions in such environments are very dynamic [84], which may be the reason for establishing relations with the large number of fungi that can adapt to frequent environment changes by changing trophic modes (endophyte-saprotroph-pathogen, Figure 6 and Figure 7). This is probably the reason why the diversity of root-associated fungal genera that influenced overall differentiation of investigated communities is the highest in this forest (Figure 4B). Similarly, soil bacterial community structures in the habitats closest to the river were most divergent from the others in the floodplain of the River Thur [85].

Soil environments in the other two habitats appeared more stable, which obviously resulted in lower number of specific fungal genera, though still with strictly distinct RAMs (Figure 4B and Figure 5B). In mixed truffle-producing forest, wide spread ECM basidiomycetous genera *Hymenogaster* sp. and *Russula* sp., both known to express varying host/habitat specificity [86,87] differentiated the RAM, together with omni-present pathogens/endophytes belonging to genera *Fusarium* sp. and *Olpidium* sp. (Figure 4B) [88]. Both of the ECM forest specific genera have been commonly detected in truffle habitats, mainly plantations [72]. The driest and most acidic, black autumn truffle-producing forest was differentiated by two ECM genera (*Humaria* sp. and *Hydnotria* sp., both ascomycetous) that have not been common in previously explored truffle grounds [72]. Common endophyte *Pezicula* sp. [88,89] and two genera (*Cladophialophora* sp. and *Humicula* sp.) that can be feeding through different trophic modes (saprotroph/ endophyte/pathotroph) were specific for this site. In single *T. macrosporum* natural site investigated for ECM diversity, Basidiomycetes (e.g., *Tomentella* sp., *Inocybe* sp.) strongly dominated the mycorrhizobiome, with *Tomentella* sp. (one of the core genera detected in present study as well) being definitely the most dominant genus, while forest specific fungal genera detected here were not detected in an Italian site [35]. The only obvious difference between Italian and the site investigated here was soil pH, which might contribute to the observed differences in ECM fungal genera. Unlike *T. magnatum* [38,39], *T. macrosporum* was relatively common in mycorrhizobiome of the natural site in Italy [35]. In our study, we could not detect it in the entire RAMs of productive sites, implying the possibility that in pooled root samples *T. macrosporum* (as well as other productive truffle species in this study) was not present in detectable quantity or due to the heterogeneity of soil the soil and root distribution.

### 4.3. Dominance of Saprotrophs and Symbiotrophs across the Seasons in Root-Associated Fungal Communities in the Studied Truffle-Producing Forests

FUNGuild analysis of the detected fungal OTUs revealed that the fungal communities were dominated by symbiotrophs and saprotrophs in all investigated forests (depending upon the sampling time points, Figure 7), while, in accordance to previous similar studies, the abundances of pathotrophic fungi were quite low [13,90,91,92]. Despite the significantly higher fungal richness detected here, trophic structures of the communities were similar to other European studied temperate forests [6,10,14,93]. However, in all other large-scale studies, ECM fungi were always strongly dominating, while, in the present study, only the autumn black truffle-producing forest resembled such trophic structure in all seasons (Figure 7). Several studies have focused on seasonal variations in ECM fungi in temperate forests [94,95,96], while little information is thus far available on the seasonality of entire fungal communities [13,97]. Most of them reported increased abundance of ECM fungi in late summer or autumn, which has also been reported previously from boreal forests [13,98,99]. Such a trend was observed in this study as well, but much more pronounced **(**Figure 7). More carbon allocation to the roots in autumn [99], with lower nutrient (presumably P) availability (Supplementary Table S1b) [27], increases the need for both partners (plants and fungi) for mycorrhiza establishment [100]. Additionally, higher ECM colonization was detected in poplars grown in soils with median levels of moisture [82,101,102], which could further explain switch to ECM domination in all investigated RAMs in autumn when the soil water content reaches the median [27]. At this time, majority of the herbs in the investigated sites have died losing the battle with the expressed drought in the upper soil layer in summer [27], which can additionally explain the increase of the dominance of ECM fungi in RAM. The water-limiting conditions favor ectomycorrhizal fungi [103], increasing symbiosis establishment to protect the plants from desiccation and starvation [104]. This natural phenomenon pretty well explains the surprising shift from strong dominance of saprotrophs to a strong dominance of ECM OTUs that especially appeared in the RAM of white truffle-producing forest on the sandy soils in this study (Figure 7B).

The white truffle-producing forest studied here has been considerably changed by human influence, in that its structure has been highly altered from the original vegetation type [84]. *Populus alba* (the only tree with almost no shrub layer) that can be assumed as the single ECM host in the habitat and is known for easily changing symbiotic partners [105], depending on the soil environment [105,106]. Despite the presence of arbuscular and ectomycorrhizal fungi, Bonito et al. (2019) [82] demonstrated that the rhizobiome of *Populus* was dominated by facultative endophytes (∼85% OTUs), most of which appeared to be cultivable and had saprotrophic activity. Our results corroborate with these findings, as root-associated communities of white poplar stand were dominated by saprotrophs in June and August, while their presence drastically dropped and were replaced by ECM fungi in the autumn (Figure 7). Highly porous, low aggregated soil with very low total N pool probably trapped in scarce OM (Supplementary Table S1) may have caused a general shortage of N availability in the upper soil layer of this site in warm seasons [7,107]. Endophytes can transform organic nitrogen to inorganic forms in the rhizosphere, making the nutrient available to their hosts [81,108,109]. High temperatures that are measured in the region in summer [27] and closeness to a water body (flooding or exposure to airborne water from river surface evaporation) may have favored the decomposition of SOM in June/August and, therefore, the establishment of root associations with fungi with high decomposing abilities that would increase plant N intake [110]. It has been confirmed that some saprotrophic fungi (also detected here) can indeed establish a facultative biotrophic relationship with roots [81,88,111]. On the other hand, alkaline soil environment (as detected here) has rarely been connected to ECM domination [7,112]. This could be to the advantage for truffles that have been connected to alkaline soils [31,35,77,79]. Nevertheless, ectomycorrhizal fungi were second dominant in RAM of poplar forest during the summer, while the presence of AMF through the entire investigated period was negligible (Figure 7). The lower abundance of AMF in our datasets could mainly be due to the well-known bias of the primer pairs towards Ascomycota and Basidiomycota [13,46,94].

It is very intriguing that majority of RAM of mixed truffle-producing forest in summer season (June and August) actually had unidentified trophic mode/ecological role, but with much higher abundance of plant pathogens than previously detected in European temperate forests (Figure 7, [13]). The high soil temperature and water table [27] that cause seasonally high soil water content and weak aeration may increase stress in plant roots and provide good conditions for pathogen development. Apart from ECM fungi, AMF were also detected in autumn RAM in this forest, though in significantly lower abundance (Figure 7). Soil nutrient status and aboveground vegetation appeared to be the main determinants of mycorrhizal fungal community composition at small geographical scales [63,113,114]. AMF presence in RAM may be induced by the presence of AM trees [115], high abundance and diversity of AM shrubs and herbs [116], as well as neutral pH [117]. As mentioned earlier, we have used general fungal primers in this work to study the overall root-associated fungal communities, the questions about the very specific roles of AMF and their diversity in this context could be addressed in another study. Additionally, some tree species such as members of *Salicaceae* and *Quercus* sp. commonly associate with both AM and ECM fungi, the latter typically becoming dominant in closed-canopy communities [106]. P availability has been connected to mycorrhizal uptake, where ECM fungi appeared more efficient in the soils with higher organically bound P, while AM fungi appeared more efficient in high inorganic P availability and in higher pH [7,106]. Increased abundance of mycorrhizal fungi in RAM of this habitat in autumn affected both mycorrhizal types equally and coincided with *T. magnatum* production maximum (Figure 7) [27].

As expected, the strongest domination of ECM fungi in RAM in all seasons (similar to European temperate forests) was detected in autumn truffle-producing forest, where the soil pH was the lowest inducing basidiomyceteous dominance [13,97,117]. ECM domination is estimated in acidic soils with lower mineral N concentrations and higher organic matter content [118,119]. Similar to our results, the study on ECM communities in *T. macrosporum* natural site revealed domination of ECM Basidiomycota, in the site in Italy where soil texture and site positioning were similar to the site explored here, but with higher pH [35].

## 5. Conclusions

Pannonian riparian forests appear to host diverse root-associated fungal communities that are strongly shaped by variation in soil conditions and characterized by seasonal patterns. The fungal communities are composed of different trophic modes and functional guilds, implying that truffle mycelia are obviously competing for C sources not only with other ECM fungi, but also with endophytes and AMF. Though our data was restricted on the root-associated mycobiome, the observed shift in the RAM community composition across the respective truffle-producing forest ecosystems indicates the interplay of the local environmental conditions, vegetation composition, and the soil conditions in shaping the soil and root inhabiting microbial communities, their co-existence, and their ecosystem functions, including truffle production, in these particular forest ecosystems.

Moreover, due to the increasing human pressure through timber harvesting in the investigated truffle-producing forest ecosystems specific soil dynamics can be destroyed which is putting these habitats and the biodiversity inhabiting them at risk. Thus, such forest ecosystems and the biodiversity therein should be taken under a special protection regime to enable sustainable truffle production/harvesting and maintenance of the co-existing root- and soil-inhabiting microbial communities and their ecosystem functions.

## Figures and Tables

**Table 1:** Selected parameters of soil fertility in each sampling plot (WTF-white truffle forest, MTF mixed truffle forest, BTF black truffle forest).

Sample	pH		SOM (%)	Available			Available (K_2_O)	CaCO_3_ (%)	Ctot (%)	Ntot (%)	C/N
	KCl	H_2_O		P_2_O_5_/jun (mg/100 g)	P_2_O_5_/aug (mg/100 g)	P_2_O_5_/nov (mg/100 g)					
WTF1	7.30	8.00	2.29	10.18	8.20	8.45	12.61	5.44	1.52	0.08	20.73
WTF2	7.50	8.10	2.27	7.46	9.45	9.58	17.12	6.49	1.83	0.09	19.71
WTF3	7.60	8.10	1.05	11.98	7.46	11.53	9.45	5.02	1.03	0.04	22.80
MTF1	5.60	6.40	3.99	9.60	20.81	3.02	21.07	-	2.16	0.23	9.39
MTF2	5.80	6.60	3.81	10.93	19.44	3.32	20.21	-	2.41	0.24	10.02
MTF3	6.30	7.00	4.78	20.95	21.86	14.89	31.82	-	2.91	0.29	10.09
BTF1	4.00	5.00	4.90	6.48	13.05	5.22	24.36	-	2.85	0.28	10.21
BTF2	3.80	5.10	4.90	5.38	12.46	4.95	11.03	-	2.28	0.23	10.08
BTF3	3.90	4.70	5.00	4.47	8.66	3.82	20.71	-	2.70	0.25	10.89

**Table 2:** Goodness-of-fit statistics (*R*^2^) for factors fitted to the two-dimensional non-metric multidimensional scaling (2D-NMDS) ordination of root-associated fungal communities in different truffles producing forest types in Serbian riparian forests. *p* values: *** = *p* < 0.001.

Variable	*R* ^2^	*p*
clay (%)	0.5737	0.001 ***
silt (%)	0.8533	0.001 ***
sand (%)	0.7169	0.001 ***
pH	0.8677	0.001 ***
OM (%)	0.7713	0.001 ***
K_2_O	0.1964	0.083
P_2_O_5_	0.2081	0.058
Distance from river (m)	0.8826	0.001 ***
Elevation (masl)	0.7499	0.001 ***
N (%)	0.7226	0.001 ***
C (%)	0.5631	0.001 ***
C:N ratio	0.7999	0.001 ***
Forest type	0.5734	0.001 ***

**Table 3:** Goodness-of-fit statistics (*R^2^*) for fungal genera fitted to the two-dimensional non-metric multidimensional scaling (2D-NMDS) ordination of root-associated fungal communities in different truffle-producing forest types in Serbian riparian forests. *p* values: ** = *p* < 0.01, *** = *p* < 0.001.

Fungal Genera	*R* ^2^	*p*
*Alternaria* sp.	0.432	0.001 ***
*Calycina* sp.	0.275	0.004 **
*Cladophialophora* sp.	0.288	0.006 **
*Cladorrhinum* sp.	0.288	0.008 **
*Cladosporium* sp.	0.341	0.004 **
*Fusarium* sp.	0.343	0.006 **
*Gibberella* sp.	0.364	0.001 ***
*Humaria* sp.	0.294	0.01 **
*Humicola* sp.	0.375	0.007 **
*Hydnotrya* sp.	0.278	0.01 **
*Hymenogaster* sp.	0.253	0.006 **
*Hymenoscyphus* sp.	0.349	0.005 **
*Mycoarthris* sp.	0.503	0.001 ***
*Olpidium* sp.	0.364	0.002 **
*Pezicula* sp.	0.287	0.009 **
*Phialophora* sp.	0.283	0.004 **
*Pyrenochaeta* sp.	0.245	0.01 **
*Russula* sp.	0.347	0.005 **
*Subulicystidium* sp.	0.571	0.001 ***

**Table 4:** Core fungal genera (defined as taxa present in 95% samples of the specific forest type) associated with white, black, and mixed truffle-producing forests and their collective percent share of the sequences from the total sequences belonging to a specific group.

White Truffle-Producing Forest		Mixed Truffle-Producing Forest		Black Truffle-Producing Forest	
*Ascochyta* sp.	**69.78%** of total sequences	*Ascochyta* sp.	**32.45%** of total sequences	Ascochyta sp.	**39.55%** of total sequences
*Exophiala* sp.		*Exophiala* sp.		*Exophiala* sp.	
*Ilyonectria* sp.		*Ilyonectria* sp.		*Ilyonectria* sp.	
*Inocybe* sp.		*Inocybe* sp.		*Inocybe* sp.	
*Tetracladium* sp.		*Tetracladium* sp.		*Tetracladium* sp.	
*Tomentella* sp.		*Tomentella* sp.		*Tomentella* sp.	
*Alternaria* sp.		*Cladophialophora* sp.		*Cladophialophora* sp.	
*Ascobolus* sp.		*Humicola* sp.		*Humicola* sp.	
*Calycina* sp.		*Hyphodiscus* sp.		*Hyphodiscus* sp.	
*Cistella* sp.		*Mortierella* sp.		*Mortierella* sp.	
*Cyphellophora* sp.		*Mycena* sp.		*Mycena* sp.	
*Fusarium* sp.		*Mycenella* sp.		*Mycenella* sp.	
*Gibberella* sp.		*Penicillium* sp.		*Penicillium* sp.	
*Hebeloma* sp.		*Russula* sp.		*Russula* sp.	
*Hymenoscyphus* sp.		*Sebacina* sp.		*Sebacina* sp.	
*Microdochium* sp.				*Athelopsis* sp.	
*Mycoarthris* sp.				*Cenococcum* sp.	
*Paraphoma* sp.				*Entoloma* sp.	
*Plectosphaerella* sp.				*Herpotrichia* sp.	
*Plenodomus* sp.				*Humaria* sp.	
*Podospora* sp.				*Menispora* sp.	
*Pyrenochaeta* sp.				*Oidiodendron* sp.	
*Rhizoglomus* sp.				*Pezicula* sp.	
*Schizothecium* sp.				*Phallus* sp.	
*Scleropezicula* sp.				*Saitozyma* sp.	
*Subulicystidium* sp.				*Trechispora* sp.	
*Tuber* sp.				*Trichoderma* sp.	

## Supplementary Materials

The following are available online at https://www.mdpi.com/2076-2607/8/9/1331/s1, Figure S1: Sample based rarefaction curves indicating the number of OTUs detected against the number of sequences based on paired-end Illumina sequencing of root-associated mycobiome different truffle production supporting habitats of Serbian riparian forests; Table S1: Texture of the investigated soils in each forest (WTF white truffle forest, MTF mixed truffle forest, BTF black truffle forest).
