# Supplementary material for: Root-Associated Mycobiome Differentiate between Habitats Supporting Production of Different Truffle Species in Serbian Riparian Forests
Source: Microorganisms. 2020 Aug 31;8(9):1331. doi: 10.3390/microorganisms8091331 (PMC7563819; doi:10.3390/microorganisms8091331)
Supplement: Supplementary file 1 [file microorganisms-08-01331-s001.pdf]

Supplementary Materials:

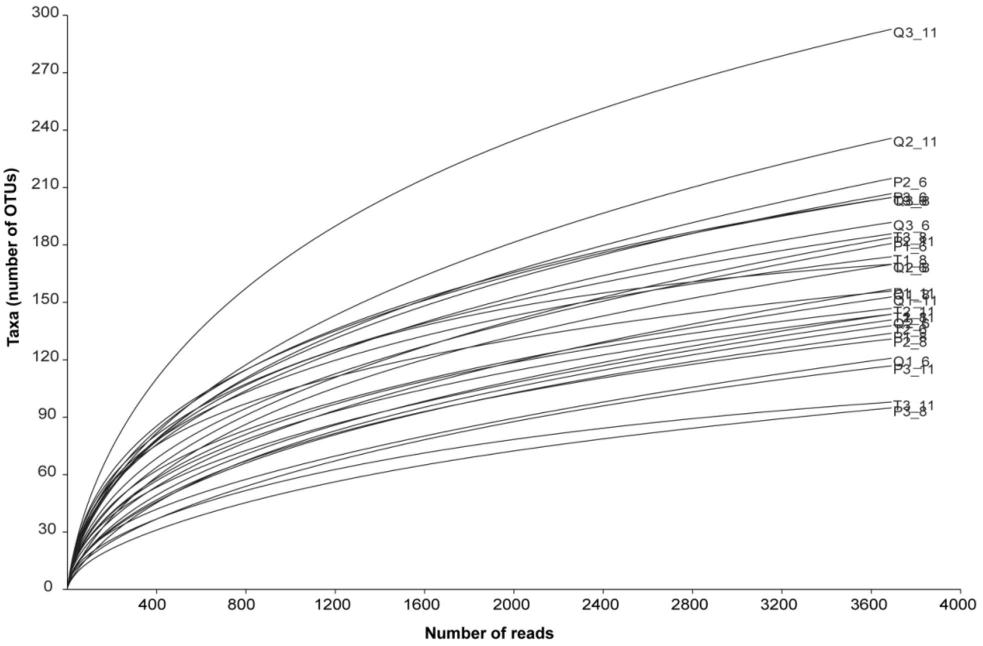

Figure S1: Sample based rarefaction curves indicating the number of OTUs detected against the number of sequences based on paired-end Illumina sequencing of root associated mycobiome different truffle production supporting habitats of Serbian riparian forests.

Table S1: Texture of the investigated soils in each forest (WTF white truffle forest, MTF mixed truffle forest, BTF black truffle forest).

sample	Large sand (>0.2mm)	Small sand (0.2–0.02mm)	Loam (0.02–0.002mm)	Clay (<0.002mm)	Total sand	Loam +Clay
	%	%	%	%	%	%
WTF	19.10	48.00	18.00	14.90	67.10	32.90
MTF	1.80	17.80	39.40	41.00	19.60	80.40
BTF	4.60	33.30	40.60	21.50	37.90	62.10
